# Clinical and psychological adjustment to prosthesis use in lower-limb amputees: focus on differences between traditional and high-technology rehabilitation

**DOI:** 10.3389/fresc.2026.1830110

**Published:** 2026-07-07

**Authors:** A. Pierobon, C. Tambussi, M. Maffoni, A. Casati, V. Neri Barbieri, B. Lops, R. Dragoni, V. Torlaschi, C. Fundarò, L. Bagnara, M. Baldini, C. Ferretti

**Affiliations:** 1 Psychology Unit of Montescano Institute, Istituti Clinici Scientifici Maugeri IRCCS, Montescano, Italy; 2 Department of Neuromotor Rehabilitation of Montescano Institute, Istituti Clinici Scientifici Maugeri IRCCS, Montescano, Italy; 3 Neurophysiopathology Unit of Montescano Institute, Istituti Clinici Scientifici Maugeri IRCCS, Montescano, Italy; 4 Department of Subacute Care of Milano Institute, Istituti Clinici Scientifici Maugeri IRCCS, Milano, Italy

**Keywords:** body image integration, high-technology rehabilitation, lower-limb amputation, multidisciplinary rehabilitation, prosthetic rehabilitation, psychological adjustment, rehabilitation outcomes

## Abstract

**Introduction:** Advanced rehabilitation technologies are increasingly being implemented in transtibial amputation, although evidence remains limited regarding whether non-immersive virtual reality systems and biofeedback provide additional benefits beyond conventional rehabilitation approaches.

**Methods:** This prospective randomized clinical trial included 21 male patients (mean age 61.7 ± 7.21 years) with transtibial amputation due to vascular disease or type 2 diabetes. Eleven participants received traditional rehabilitation, while ten underwent a combined protocol integrating conventional physiotherapy with D-Wall technology over a 3-week inpatient program.

**Results:** Across the entire sample, functional outcomes related to autonomy and prosthetic mobility improved from baseline to discharge, including the Barthel Index, Functional Independence Measure, and Amputee Mobility Predictor, independent of rehabilitation condition. A non-significant trend toward reduced depressive symptoms was also observed. Baseline psychological characteristics were associated with rehabilitation outcomes. Higher self-efficacy was associated with lower depressive symptoms, anxiety, and body image distress, as well as higher prosthetic mobility. Similarly, better prosthetic adaptation at discharge was associated with lower psychological distress and higher quality of life and functional independence. Exploratory within-group analyses suggested that, in the technology-enhanced group, improvements in body image were associated with reductions in anxiety, while reductions in depressive symptoms were associated with higher functional independence. In the traditional rehabilitation group, reductions in anxiety were associated with improved perceived quality of life.

**Discussion:** Overall, technology-enhanced rehabilitation was not associated with superior functional outcomes compared with traditional rehabilitation in this exploratory sample. However, exploratory findings suggest potential associations with psychological and perceptual processes related to emotional adjustment and body image during rehabilitation. These preliminary observations should be confirmed in adequately powered studies.

## 1 Introduction

Amputation is a surgical procedure involving the partial or total removal of a limb or body segment, typically performed due to severe pathologies, trauma, infections, or neoplasms. Although upper-limb amputations also occur, lower-limb amputation is the most common and represents one of the leading causes of disability worldwide (1). Non-traumatic amputation frequently results from complex conditions involving vascular, metabolic, infectious, and social factors, and its incidence is steadily increasing in relation to population ageing and the growing prevalence of diabetes mellitus and peripheral vascular diseases (2, 3).

Diabetes mellitus is a chronic metabolic disease characterized by persistent hyperglycemia due to impaired insulin secretion and/or action. Its global prevalence has increased substantially in recent decades (4). Type 2 diabetes mellitus (T2DM) accounts for approximately 90%–95% of cases and is a major contributor to lower-limb amputation risk, particularly in the presence of long-standing disease and comorbid vascular complications (3, 5). In diabetic foot ulcers, amputation risk may reach 31%, with additional predictors including male sex, hypertension, and renal disease (3). Socioeconomic disparities further increase risk, highlighting the role of access to care and broader systemic determinants of health (2, 6).

Beyond its medical implications, limb amputation represents a traumatic and transformative event that affects identity, body experience, and psychological functioning. It requires extensive adaptation to new physical conditions, daily activities, and social roles, with consequences that may persist over time and significantly affect quality of life (7–9). Common psychological outcomes include depressive and anxiety symptoms, particularly social anxiety related to body exposure and perceived stigma (8, 9). Body image disturbance is also a central component of adjustment, as patients may experience the limb loss as a disruption of bodily integrity, often accompanied by emotional reactions such as sadness, fear, anger, and denial (8, 33).

Psychological adjustment is further shaped by new practical challenges associated with the new clinical condition: for example, patients need to relearn basic activities of daily living, which become complex and effortful following amputation (10). However, adaptation processes may be facilitated by adequate social and family support, goal redefinition, and psychological interventions targeting resilience, self-efficacy, and coping resources (6, 11).

Within this context, prosthetic rehabilitation plays a central role in functional and psychosocial recovery: the prosthesis is not only a mechanical device but also a means of restoring bodily continuity and participation in daily life (8). The prosthetic process is multidisciplinary and includes clinical evaluation, progressive training, and follow-up for adaptation to residual limb changes (12). Outcomes depend on individual (age, comorbidities, motivation), environmental (family and social context), and organizational factors (access to specialized services) (10, 36). However, prosthetic adherence remains a challenge, with discontinuation rates of 30%–50% within the first year due to pain, technical difficulties, or insufficient support (8, 13). For these reasons, rehabilitation programs should be early, individualized, and multidisciplinary, integrating physical and psychological components to optimize functional recovery and quality of life (3, 36). In amputee patients, high-technology rehabilitation has shown potential benefits in improving prosthetic adaptation. Gait-support technologies, such as body-weight, supported treadmill training, allow for safe and progressive ambulation practice, reducing joint stress and fall risk. Meanwhile, biofeedback systems help patients develop awareness of postural alignment and compensatory mechanisms, thereby reducing the risk of muscular overload (14). Recent studies further suggest that advanced rehabilitation programs may be associated with improvements in autonomy, treatment adherence, and patient satisfaction, possibly due to their capacity to foster more active patient involvement, motivation, and self-efficacy throughout the rehabilitation process (15, 16). Alongside these technological advances, virtual reality (VR) has attracted growing attention as an innovative rehabilitation strategy aimed at supporting personalized functional recovery, reintegration into daily life, and maintaining quality of life. Furthermore, non-immersive VR systems have been proposed as accessible tools to enhance motivation, autonomy, and active participation within rehabilitation pathways while complementing conventional therapeutic approaches (34, 35).

In this context, the present randomized prospective clinical study was designed as an exploratory investigation of a traditional rehabilitation program and a combined intervention integrating conventional rehabilitation and non-immersive virtual reality-based technology in patients with transtibial amputation. The primary aim was to explore the feasibility and potential effects of the technology-enhanced intervention on functional outcomes, psychological adaptation, quality of life, and emotional well-being compared with traditional rehabilitation alone. Given the exploratory nature of the study and the limited sample size, no confirmatory efficacy hypotheses were formulated. Rather, the study sought to generate preliminary evidence and estimate the magnitude of potential effects to inform future adequately powered clinical trials. Secondary aims included the exploration of associations between baseline psychological characteristics and rehabilitation outcomes, the identification of individual patterns of psychological and functional adaptation during prosthetic rehabilitation, and the assessment of usability and acceptability of the technological intervention, with particular attention to its perceived impact on motivation, autonomy, and self-perception. Given the exploratory nature of the study and the limited sample size, no confirmatory efficacy hypotheses were formulated. Instead, the study was intended to generate preliminary evidence and estimate effect sizes to inform future adequately powered clinical trials.

## 2 Materials and methods

### 2.1 Participants

The study sample consisted of 21 adult patients with lower-limb amputation due to vascular causes or type 2 diabetes mellitus, all admitted to the IRCCS Maugeri Institute in Montescano to begin a multidisciplinary rehabilitation program. All participants were in the phase of prosthetic adaptation and were engaged in a therapeutic pathway aimed at promoting both functional and psychological recovery. The study is part of a larger ongoing project at IRCCS Maugeri, Montescano (Clinical Trial ID: NCT06471855; Ethical Committee approval No. 2768, 31 May 2023).

Participants were selected based on specific clinical and cognitive criteria: they had to be younger than 80 years of age, have completed the surgical phase, and be in general health conditions compatible with the initiation of an individualized rehabilitation program. At study entry, information regarding ongoing psychological counseling, psychotherapy, or psychiatric treatment outside the rehabilitation program was also collected. None of the enrolled participants reported receiving external psychological or psychiatric support during the study period.

The patients were recruited during the rehabilitation phase, which starts right after the recovery of the surgical wound: time span between operation and hospitalization is at least 2 months and not more than 3 months.

Participants were allocated to the experimental or control group using a pre-generated randomization list. Specifically, a sequence of binary codes (0 = control group; 1 = experimental group) was created prior to participant enrollment and recorded in an Excel file. As eligible patients were consecutively admitted, they were assigned to groups according to the next available code in the sequence.

Due to the nature of the rehabilitation intervention, blinding of participants and clinicians was not feasible. No formal allocation concealment procedure was implemented. However, outcome assessments were conducted using standardized instruments to reduce potential assessment bias.

For all patients, the prosthesis was based on the Ottobock Amparo System, a suspension system with a one-way vacuum valve liner and a knee pad, associated with 1C11 multiaxial carbon foot.

Of the 21 participants, 10 were assigned to the experimental group, which received a combined rehabilitation intervention integrating traditional techniques with high-technology devices based on non-immersive virtual reality. The remaining 11 patients constituted the control group and received only traditional rehabilitation treatment. The duration of the intervention was identical for both groups (90 min).

All participants received comprehensive information regarding the aims, procedures, and implications of the study and provided written informed consent in accordance with current ethical and legal standards.

Given the limited sample size, this study should be considered exploratory in nature. No *a priori* sample size calculation was performed, as the sample reflects the number of eligible patients consecutively admitted during the study period. Therefore, the results should be interpreted with caution and considered preliminary.

### 2.2 Inclusion and exclusion criteria

Participants were selected based on clearly defined clinical, functional, and cognitive criteria. The inclusion criteria were: age below 80 years, presence of a transtibial amputation, and admission to the IRCCS Istituti Clinici Scientifici Maugeri in Montescano to undergo a rehabilitation program during the prosthetic adaptation phase. Enrollment in the study required the ability to actively participate in the therapeutic pathway and a clinically stable condition suitable for initiating rehabilitation.

Patients with severe clinical conditions, such as acute neoplasms or other non-stabilized chronic diseases, that could interfere with participation in the program were excluded. Additional exclusion criteria included a history or current presence of central nervous system disorders (e.g., stroke or vascular brain injury), joint diseases limiting motor activity, and significant visuoperceptual deficits.

Individuals with non-Italian educational backgrounds (whose comprehension of self-report measures might be inadequate), those with illiteracy or functional illiteracy, and patients who lacked sufficient motivation or declined assessment were also excluded. Finally, patients with severe, previously unreported psychiatric disorders and those with impaired cognitive functioning, as assessed using the Mini-Mental State Examination [MMSE, (17, 18)] with scores equal to or below 26/30, were excluded.

### 2.3 Measures

A battery of standardized instruments was administered for quantitative evaluation. The tests were conducted during hospitalization, primarily in a self-report format, with assistance provided by the researcher when patients required support in completing the measures. This procedure ensured that all participants, according to their level of autonomy, were able to understand and complete the instruments appropriately.

Some assessments were administered only at baseline, others only at discharge, and some at both time points, allowing for the observation of changes from the initial phase. The instruments were selected based on their sensitivity to short-term variations, whereas measures assessing more stable characteristics were not repeated. Additionally, specific tools related to the prosthetic experience and the rehabilitation process were administered exclusively at the end of treatment, when the prosthesis was in use and the intervention had been completed.

The measures, constructs assessed, score ranges, and time points of administration are summarized in Table 1. A detailed description of the TAPES and ASoNA subscales is provided in Table 2.

**Table 1:** Psychological, cognitive and functional tests used during the study.

Scale	Construct	Range	Score interpretation (↑ high ↓ low)		*T*_0_	*T*_1_
			Score	Construct		
Psychological scales						
MMSE	Global cognitive functioning	0–30	↑	↑	✓	X
EQ-VAS	Health-related quality of life	0–100	↑	↑	✓	✓
GAD-7	Anxiety symptoms	0–21	↑	↑	✓	✓
PHQ-9	Depressive symptoms	0–27	↑	↑	✓	✓
ASonA	Adherence and self-efficacy	–	↑	↑	✓	X
BIS	Body image distress	0–30	↑	↑	✓	✓
TAPES	Adjustment to the prosthesis	–	↑	↑	X	✓
TARPP-Q	Perception of technological rehabilitation	–	↑	↑	X	✓
Functional scales						
Barthel	Autonomy in ADLs	0–100	↑	↑	✓	✓
Morse	Fall risk	0–51	↑	↑	✓	✓
FIM	Level of disability and need for assistance in ADLs	18–126	↑	↑	✓	✓
AMP	Functional mobility in amputees	0–42	↑	↑	✓	✓

Summary of the principal assessment instruments used to evaluate cognitive functioning, emotional well-being, self-efficacy, body image, prosthetic adaptation, patient perception of technology-assisted rehabilitation, and functional autonomy. MMSE, mini-mental state examination (17, 18); EQ VAS, EuroQoL visual analogue scale (19, 20); GAD-7, generalized anxiety disorder-7 (21); PHQ-9, patient health questionnaire-9 (22); ASonA, antecedents and self-efficacy on adherence; BIS, body image scale (23–25); TAPES, trinity amputation and prosthesis experience scales (26, 27); TARPP-Q, technology-assisted rehabilitation patient perception questionnaire (28); Barthel, modified Barthel index (29); Morse, Morse fall scale (30); FIM, functional independence measure (31); AMP, amputee mobility predictor (32).

**Table 2:** Overview of TAPES and ASonA subscales, score ranges, and psychological constructs assessed.

Scale	Subscale	Range	Construct
TAPES			
	A.G. TAPES General adjustment	1–5	Assesses the overall degree to which the person perceives having adapted to the condition of amputation and to the use of the prosthesis, considering psychological well-being and acceptance of the new situation
	A.S. TAPES Social adjustment	1–5	Measures the impact of amputation on the patient's social life, such as perceived social acceptance, integration within relational contexts, and the level of embarrassment or social avoidance
	A.L. TAPES Adjustment to limitations	1–5	Assesses the extent to which the patient perceives being able to cope with the physical and functional limitations related to amputation, including the sense of control and self-efficacy regarding their condition
	S.E. TAPES Aesthetic satisfaction	1–5	Explores the aesthetic perception of the prosthesis, that is, how satisfied the patient is with its appearance and with the harmony between the prosthesis and their body
	S.P. TAPES Weight satisfaction	1–5	Assesses satisfaction with the weight of the prosthesis, meaning the extent to which the prosthesis is perceived as “heavy” or “light” in everyday life
	S.F. TAPES Functional satisfaction	1–5	Assesses the level of satisfaction with the functional performance of the prosthesis, such as comfort, ease of use, and its usefulness in daily life
ASoNA			
	A-ASonA Antecedents	0–24	Assesses the degree of illness acceptance and the support perceived from family members and friends
	SE-ASonA Self-Efficacy	0–24	Assesses the individual's confidence in their ability to manage the illness and adhere to medical prescriptions
	Aff-ASonA Positive and negative affectivity	0–24	Assesses the emotional state related to the illness

The table summarizes the subdimensions of the trinity amputation and prosthesis experience scales (TAPES) and the antecedents and self-efficacy on adherence scale (ASonA), including score ranges and the primary psychological or functional domains evaluated. Higher scores indicate greater levels of the construct assessed within each subscale.

#### 2.3.3 Rehabilitation therapy

##### 2.3.3.1 Traditional motor therapy

Traditional motor therapy represents the foundational component of the rehabilitation pathway for individuals with lower-limb amputation. It includes targeted exercises aimed at improving joint mobility, muscle strengthening, and functional training, with a specific focus on balance, postural stability, and gait. The primary objective is to enhance functional independence, increase exercise tolerance, and prepare the patient for prosthesis use.

The treatment is delivered daily for a total of 60 min under the supervision of experienced physiotherapists, in accordance with the Individualized Rehabilitation Project (PRI). The intervention spans three consecutive weeks of inpatient rehabilitation at IRCCS Maugeri in Montescano, with five weekly sessions of 90 min each. For patients assigned to the traditional rehabilitation group, each session includes 60 min of motor physiotherapy and 30 min dedicated to clinical supervision, monitoring, or supportive consultations.

##### 2.3.3.2 High-technology rehabilitation: D-Wall

In addition to the traditional intervention, the present study incorporated advanced technological tools for the experimental group, with the aim of complementing the rehabilitation pathway through devices capable of increasing engagement and providing immediate, objective feedback. The technology employed was the D-Wall system (TecnoBody SRL, Italy), an interactive digital wall equipped with a 3D camera and motion sensors, enabling real-time assessment and training of postural control, gait initiation, balance, and spatial orientation through guided exercises.

While standing in front of the digital wall, patients interact with a visual interface displaying an avatar and graphical indicators, providing real-time biofeedback during movement execution. The absence of markers or wearable devices ensures a natural and non-invasive experience. The system allows for individualized training protocols based on the patient's functional level and offers precise monitoring of progress over time.

Patients in the experimental group received the same daily treatment duration as those in the control group: 60 min of traditional motor rehabilitation supplemented by 30 min of D-Wall–based training, always supervised by a physiotherapist trained in the use of the device. Sessions were carried out 5 days per week over a continuous 3-week inpatient period.

The integration of technological systems such as the D-Wall represents an important innovation in post-amputation rehabilitation, supporting not only the optimization of motor parameters but also potential improvements in psychological dimensions such as body image, self-efficacy, and treatment adherence.

### 2.4 Procedure

Patients admitted to the rehabilitation unit of IRCCS Maugeri in Montescano were invited to participate in the research project following a routine psychological interview conducted by the facility's Clinical Psychologists/Psychotherapists. During this initial meeting, the study objectives, procedures, timeline, data anonymization process, and the voluntary nature of participation, including the option to withdraw at any time without consequences for the therapeutic pathway, were clearly explained.

Patients deemed potentially eligible underwent preliminary cognitive screening using the Mini-Mental State Examination (MMSE). Those meeting the inclusion criteria were subsequently invited for a second appointment to sign the informed consent and privacy agreement. Data collection commenced only after signed consent was obtained.

**Figure 1:**
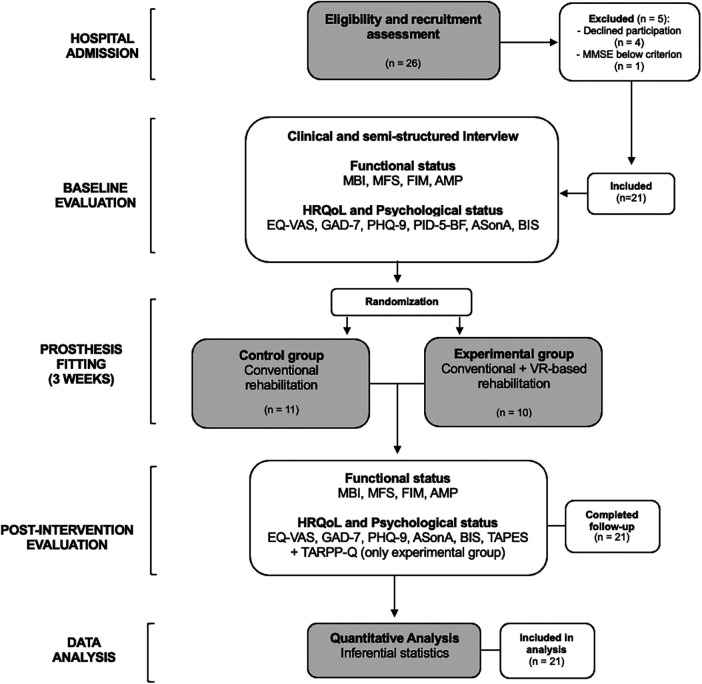
Flow diagram of participant recruitment, study procedure, and assessment timeline.

The overall procedure consisted of several phases, as outlined in the flowchart below (Figure 1). Upon hospital admission, participants completed a baseline assessment, which included a clinical interview and the administration of psychometric and functional measures.

The rehabilitation program was designed according to the physiatric Diagnostic, Therapeutic and Assistance Plan (PDTA), considering the goals established in the Individualized Rehabilitation Project (PRI) and its corresponding individualized rehabilitation program. The minimum duration of the rehabilitation treatment was 3 weeks. During hospitalization, all patients took part in psychoeducational and therapeutic sessions led by different healthcare professionals—including nutritionists, physiotherapists, physiatrists, and psychologists—and participated in motor rehabilitation activities.

Depending on group allocation, patients received either traditional rehabilitation (control group) or a combined protocol including both traditional rehabilitation and high-technology training (experimental group).

At the end of the rehabilitation program, participants completed a follow-up assessment involving the administration of the same psychometric and functional instruments.

The study procedure is illustrated in Figure 1.

### 2.5 Data analysis

First, descriptive statistics were computed for all sociodemographic, clinical, functional, and psychological variables included in the sample. The normality of distributions was assessed using the Shapiro–Wilk test, while homogeneity of variances between groups was evaluated using Levene's test. Baseline differences were examined using independent-samples *t*-tests for continuous variables and frequency comparisons for categorical variables.

To examine overall change over time across the full sample, pre–post (*T*_0_–*T*_1_) comparisons were performed on the main functional and psychological measures. The effect of rehabilitation type was subsequently investigated using a repeated-measures ANCOVA with a 2 × 2 design (Time × Group), including covariates that will be described in the following sections.

Associations between changes over time were assessed using correlations computed on change scores (Δ), analyzed both for the entire sample and separately within each treatment group. To explore the predictive value of key psychological and functional indicators, simple linear regression models were first estimated, followed by multiple regression models including additional predictors that will be detailed later.

Finally, model robustness was evaluated through *post-hoc* power analyses and sensitivity analyses, while coefficient stability was further examined using bootstrap procedures (5,000 resamples) and leave-one-out validation.

All analyses were conducted using Jamovi (version 2.6.45).

## 3 Results

The sample consisted of 21 transtibial amputee patients, all males. The mean age was 61.7 years (SD = 7.21). Eleven participants (52.4%) underwent a traditional rehabilitation program, while 10 (47.6%) received a high-technology rehabilitation program.

Normality tests (Shapiro–Wilk) confirmed a normal distribution for most variables, with the exception of years of education (*p* = .017). Levene's tests for homogeneity of variances yielded non-significant results (*p* > .05), confirming comparability between groups. Baseline between-group comparisons were performed using independent-samples *t*-tests for normally distributed continuous variables and Mann–Whitney *U* tests for non-normally distributed variables. Categorical variables did not differ significantly, further supporting sample homogeneity across the two groups. The only significant baseline difference concerned years of education, which were higher in the high-technology group (*t*= −2.14, *p* = .046, *d* = −0.94). Baseline characteristics and between-group comparisons are reported in Table 3 (continuous variables) and Table 4 (categorical variables).

**Table 3:** Baseline demographic, functional and psychological characteristics of the study sample.

Variable	Traditional (*n* = 11)	High-tech (*n* = 10)	Test (*t*/*U*)	*p*-value
	M ± SD	M ± SD		
Demographics				
Age (years)	62.0 ± 6.41	61.3 ± 8.20	*t* = 0.07	.947
Weight (kg)	75.0 ± 10.6	80.2 ± 11.7	*t* = 1.10	.285
Height (cm)	173 ± 6.7	177 ± 5.9	*t* = −1.50	.150
BMI	24.7 ± 4.5	26.1 ± 5.3	*t* = 1.27	.220
Years of education	8.9 ± 2.6	11.7 ± 3.5	*U* = −2.14	.046*
Functional	28.9 ± 1.2	28.8 ± 1.35	*t* = 0.06	.954
Barthel index (*t*0)	25.0 ± 17.6	26.0 ± 16.9	*t* = −0.13	.898
Morse (*t*0)	95.0 ± 8.2	95.0 ± 7.8	*t* = 0.00	1.000
FIM (*t*0)	11.0 ± 4.8	13.4 ± 6.0	*t* = 1.18	.251
AMP (*t*0)	66.5 ± 23.2	76.2 ± 24.4	*t* = −0.92	.367
Psychological				
MMSE	67.4 ± 15.8	68.7 ± 15.4	*t* = −0.25	.805
EQ-VAS (t0)	3.1 ± 4.0	5.4 ± 6.2	*t* = 34.5	.152
GAD-7 (t0)	5.3 ± 6.0	5.8 ± 6.7	*t* = 52.0	.859
PHQ-9 (t0)	6.0 ± 7.3	7.0 ± 6.0	*t* = 0.37	.715
BIS (t0)	2.0 ± 2.3	4.2 ± 2.5	*t* = 25.5	.035
A-ASoNA	17.9 ± 5.0	19.3 ± 5.9	*t* = −0.61	.546
SE-ASoNA	21.1 ± 9.2	23.7 ± 8.9	*t* = −0.57	.571
Aff-ASoNA	91.5 ± 30.4	100.1 ± 31.8	*t* = 0.12	.903
Tot-ASoNA	62.0 ± 6.41	61.3 ± 8.20	*t* = 0.07	.947

Continuous variables are presented as mean ± standard deviation (M ± SD). Between-group comparisons were conducted using independent-samples *t*-tests for normally distributed variables and Mann–Whitney *U* tests where appropriate. The “Test” column reports *t* or *U* statistics. Statistically significant differences are indicated in bold and marked with an asterisk (*), corresponding to *p* < 0.05. Higher scores indicate greater levels of the measured construct for all scales, except where otherwise specified. MMSE, mini-mental state examination; EQ-VAS, EuroQoL visual analogue scale; GAD-7, generalized anxiety disorder-7; PHQ-9, patient health questionnaire-9; BIS, body image scale; ASoNA, antecedents and self-efficacy on adherence; TAPES, trinity amputation and prosthesis experience scales; Barthel, modified Barthel index; Morse, Morse fall scale; FIM, functional independence measure; AMP, amputee mobility predictor.

**Table 4:** Baseline demographic, functional and psychological characteristics of the study sample.

Variable	Traditional (*n* = 11)	High-tech (*n* = 10)	Test (*χ*^2^)	*p*-value
Gender				
Male	11 (100.0%)	10 (100.0%)	–	–
Working status				
Retired	7 (63.6%)	5 (50.0%)	*χ*^2^ = 2.49	.646
Unemployed	1 (9.1%)	1 (10.0%)		
Homemaker	0 (0.0%)	1 (10.0%)		
Employed	3 (27.3%)	2 (20.0%)		
Other	0 (0.0%)	1 (10.0%)		
Marital status				
Single	5 (45.5%)	5 (50.0%)	*χ*^2^ = 1.43	.698
Married	4 (36.4%)	3 (30.0%)		
Divorced/Separated	2 (18.2%)	1 (10.0%)		
Widowed	0 (0.0%)	1 (10.0%)		
Living condition				
Alone	4 (36.4%)	2 (20.0%)	*χ*^2^ = 3.49	.624
With partner	3 (27.3%)	2 (20.0%)		
With partner and children	1 (9.1%)	1 (10.0%)		
With children	0 (0.0%)	1 (10.0%)		
With other relatives	2 (18.2%)	4 (40.0%)		
Assisted facilities	1 (9.1%)	0 (0.0%)		
Caregiver				
Spouse	2 (18.2%)	2 (20.0%)	*χ*^2^ = 3.96	.555
Son/Daughter	1 (9.1%)	2 (20.0%)		
Parents	0 (0.0%)	2 (20.0%)		
Other family member	4 (36.4%)	2 (20.0%)		
Non-family caregiver	1 (9.1%)	1 (10.0%)		
None	3 (27.3%)	1 (10.0%)		
Comorbidity				
Type 2 DM	9 (81.8%)	4 (40.0%)	*χ*^2^ = 4.55	.103
PAD	0 (0.0%)	2 (20.0%)		
Type 2 DM + PAD	2 (18.2%)	4 (40.0%)		
Risk factor: smoking				
No	2 (18.2%)	4 (40.0%)	*χ*^2^ = 4.61	.203
Yes	2 (18.2%)	3 (30.0%)		
Former smoker	7 (63.6%)	2 (20.0%)		
Risk factor: alcohol				
No	5 (45.5%)	6 (60.0%)	*χ*^2^ = 1.05	.593
Yes	3 (27.3%)	1 (10.0%)		
Past use	3 (27.3%)	3 (30.0%)		

Categorical variables are presented as absolute frequencies (*n*) and percentages (%) of the total group size. Between-group comparisons were conducted using the Chi-square test (*χ*^2^). For each variable, the values represent the number of subjects falling into each category relative to the respective group's total (*n*); for example, in the Traditional group, 7 out of 11 participants (63.6%) are retired. DM, diabetes mellitus; PAD, peripheral artery disease.

Given the limited sample size, regression models, subgroup analyses, and associative findings should be considered exploratory in nature and interpreted with caution.

**Figure 2:**
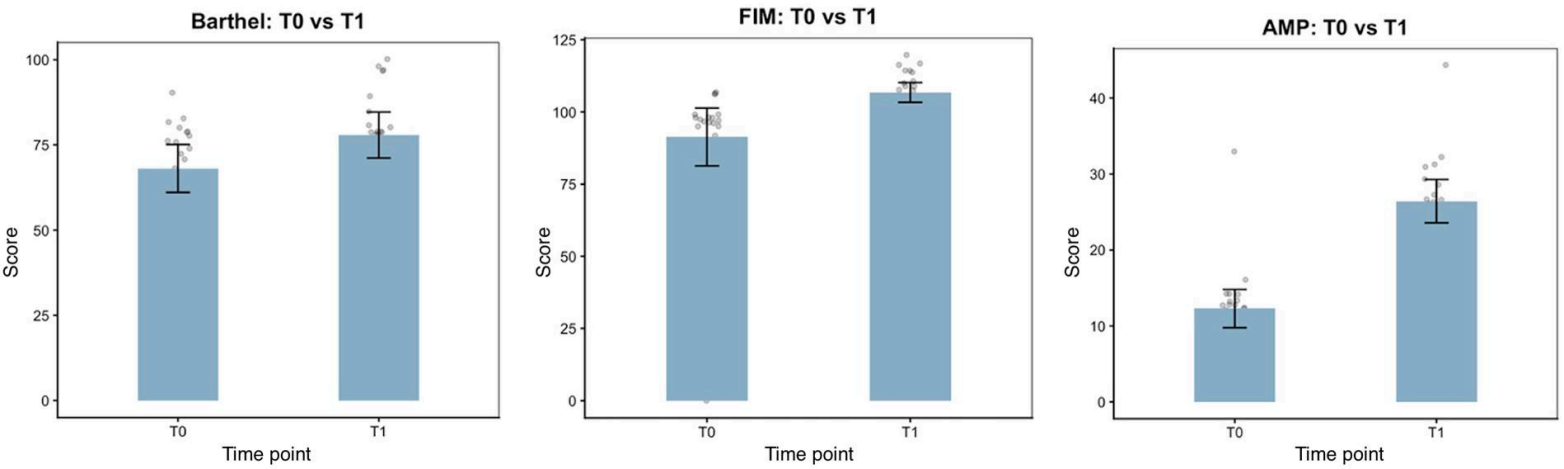
Modified Barthel Index (Barthel), functional independence measure (FIM), and amputee mobility predictor (AMP) between baseline (*T*_0_) and post-rehabilitation (*T*_1_). Bars represent mean values for each assessment at the two time points, with error bars indicating the standard error of the mean. Individual participant scores are displayed as grey dots to illustrate within-group variability. Higher scores on all three measures indicate better functional independence or mobility performance.

The pre–post comparison (*T*_0_–*T*_1_) for the entire sample showed significant improvements in functional autonomy and prosthetic adaptation. The Barthel Index demonstrated a significant increase (*p* = .001, *r* = 1.00). The FIM scale also showed a significant improvement (*p* < .001, *r* = 1.00), as did the AMP scale (*p* < .001, *r* = 1.00). The very large effect size estimates observed for some pre–post comparisons should be interpreted cautiously, as rank-based non-parametric statistics may yield ceiling-level effect sizes in small samples when all participants show changes in the same direction. Therefore, these values likely reflect the consistency of improvement across participants rather than the magnitude of the clinical effect itself. The PHQ-9 questionnaire showed a trend toward improvement (*p* = .057, *r* = .53) (see Table 5 and Figure 2).

**Table 5:** Within-group changes in functional and psychological outcomes from baseline (*T*0) to discharge (*T*1).

Variable	Mean (*t*0)	Mean (*t*1)	Mean difference (*t*0–*t*1)	Test	*p*	Effect size (*r*)
Barthel	68.1	82.6	−14.50	*W* = 0	**.** **001**	−1.000*
Morse	25.5	13.0	+12.50	*W* = 15	.396	+0.429
FIM	95.0	107.0	−12.00	*W* = 0	**<.** **001**	−1.000*
AMP	12.3	26.8	−14.50	*W* = 0	**<.** **001**	−1.000*
EQ-VAS	71.4	76.4	−5.00	*W* = 64	.356	−0.251
PHQ-9	5.57	3.57	+2.00	*W* = 117	.057	+0.529
GAD-7	4.52	3.52	+1.00	*W* = 84.5	.407	+0.243
BIS	6.48	4.98	+1.50	*W* = 64.5	.468	+0.229

Values represent mean scores at baseline (*t*0) and post-intervention (*t*1), the mean difference between time points, the Wilcoxon signed-rank test statistic (*W*), the corresponding *p*-value, and the effect size (*r*). Negative mean differences indicate improvement for scales where higher scores reflect better functioning (e.g., Barthel Index, FIM, AMP, EQ-VAS), whereas positive mean differences indicate improvement for scales where lower scores reflect fewer symptoms or risks (e.g., Morse Fall Scale, PHQ-9, GAD-7, BIS). Statistically significant differences are indicated in bold and marked with an asterisk (*), corresponding to *p* < 0.05. Barthel, modified Barthel index; Morse, morse fall scale; FIM, functional independence measure; AMP, amputee mobility predictor; EQ-VAS, EuroQoL visual analogue scale; GAD-7, generalized anxiety disorder-7; PHQ-9, patient health questionnaire-9; BIS, body image scale.

Correlations between change scores (Δ) over time showed, for the entire sample, a significant positive association between the change in anxiety symptoms (GAD-7) and the change in depressive symptoms (PHQ-9) (*ρ* = .56, *p* = .008).

Exploratory subgroup analyses were performed separately for the traditional and high-technology rehabilitation groups. In the traditional rehabilitation group, the reduction in anxiety symptoms (GAD-7) was associated with a decrease in depressive symptoms (PHQ-9) (*ρ* = .67, *p* = .025) and with an increase in perceived quality of life (EQ-VAS) (*ρ* = .66, *p* = .026). In the high-technology group, the reduction in depressive symptoms (PHQ-9) was correlated with an improvement in functional independence (FIM) (*ρ* = .65, *p* = .042), whereas the reduction in anxiety symptoms (GAD-7) was associated with an improvement in body image (BIS) (*ρ* = .75, *p* = .013).

Exploratory regression analyses were conducted to examine the relationships between change scores.

**Figure 3:**
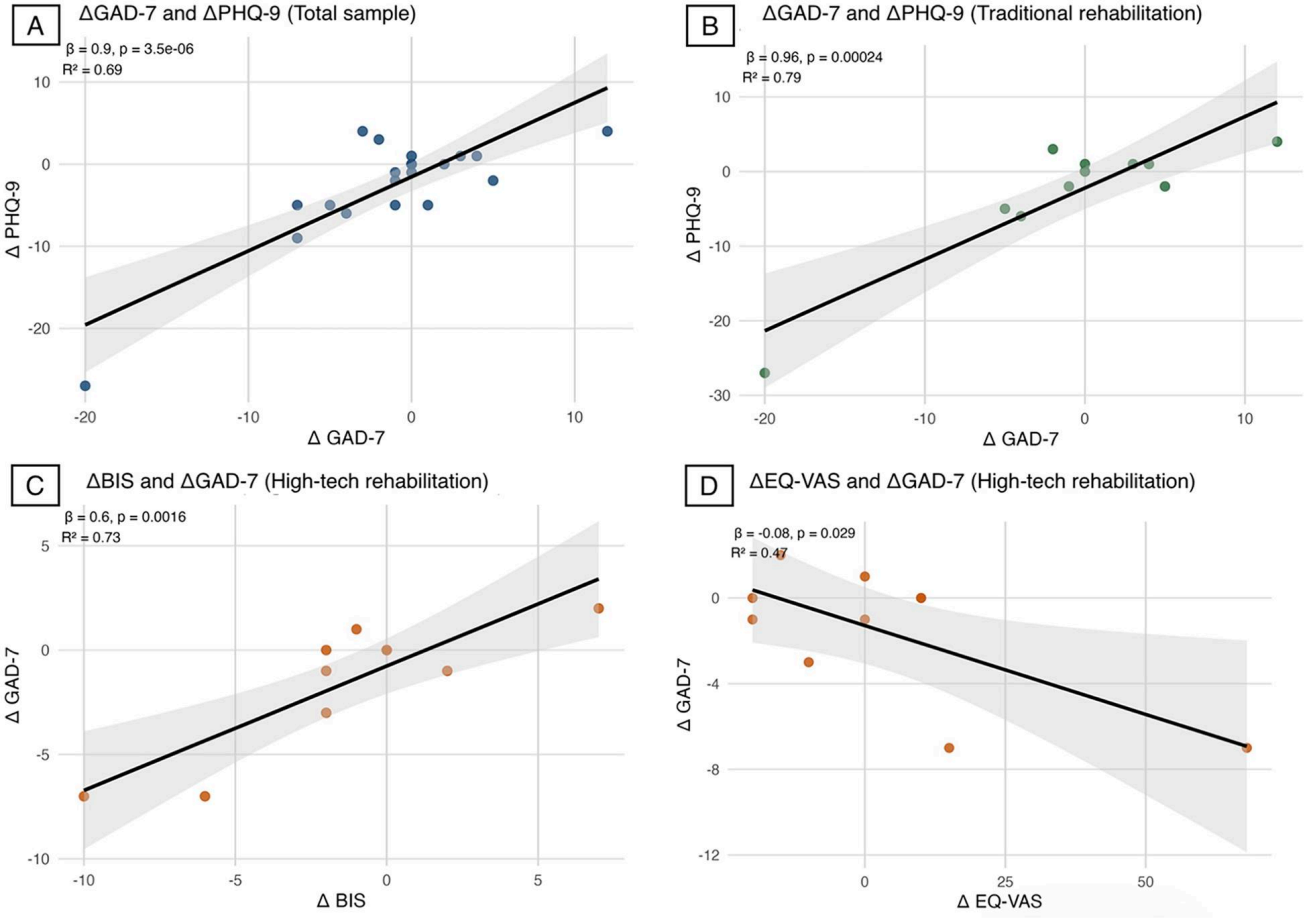
Scatterplots illustrate linear relationships between change scores. **(A)** ΔGAD-7 and ΔPHQ-9 in the total sample. **(B)** ΔGAD-7 and ΔPHQ-9 in the traditional rehabilitation group. **(C)** ΔBIS and ΔGAD-7 in the high-tech rehabilitation group. **(D)** ΔEQ-VAS and ΔGAD-7 in the high-tech rehabilitation group. Solid lines represent linear regression models with 95% confidence intervals. EQ VAS, EuroQoL visual analogue scale; GAD-7, generalized anxiety disorder-7 items; PHQ-9, patient health questionnaire-9 items; BIS, body image scale.

It showed that the change in anxiety symptoms (GAD-7) was significantly associated with the change in depressive symptoms (PHQ-9) in the overall sample [*F*_(1,19)_ = 41.6, *p* < .001, *R*^2^ = .69, *β* = .83], with an even stronger association in the group undergoing traditional rehabilitation [*F*_(1,9)_ = 34.3, *p* < .001, *R*^2^ = .79]. In the experimental group, improvement in body image perception (BIS) was significantly associated with a reduction in anxiety symptoms (GAD-7) [*F*_(1,8)_ = 21.8, *p* = .002, *R*^2^ = .73], whereas increased perceived quality of life (EQ-VAS) was associated with a significant reduction in anxiety symptoms (GAD-7) [*F*_(1,8)_ = 7.08, *p* = .029, *R*^2^ = .47]. Linear relationships between change scores are illustrated in Figure 3. The ASoNA dimensions, acceptance of illness-related limitations (A-ASoNA), perceived self-efficacy (SE-ASoNA), and emotional response to one's health condition (Aff-ASoNA), showed significant baseline associations with post-treatment psychological and functional outcomes. Acceptance (A-ASoNA) was negatively correlated with depressive symptoms (PHQ-9) (*ρ* = −.581, *p* = .006) and with body image perception (BIS) (*ρ* = −.577, *p* = .006). Self-efficacy (SE-ASoNA) was negatively correlated with depressive symptoms (PHQ-9) (*ρ* = −.503, *p* = .020), anxiety symptoms (GAD-7) (*ρ* = −.454, *p* = .039), and body image perception (BIS) (*ρ* = −.521, *p* = .016), and positively correlated with prosthetic mobility and functionality (AMP) (*ρ* = .544, *p* = .011) and perceived quality of life (EQ-VAS) (*ρ* = .460, *p* = .036). The emotional response to the health condition (Aff-ASoNA) was negatively correlated with body image perception (BIS) (*ρ* = −.606, *p* = .004). Finally, the total ASoNA score (ASoNA tot) was associated with lower levels of depressive symptoms (PHQ-9) (*ρ* = −.583, *p* = .006) and better body image perception (BIS) (*ρ* = −.599, *p* = .004).

**Figure 4:**
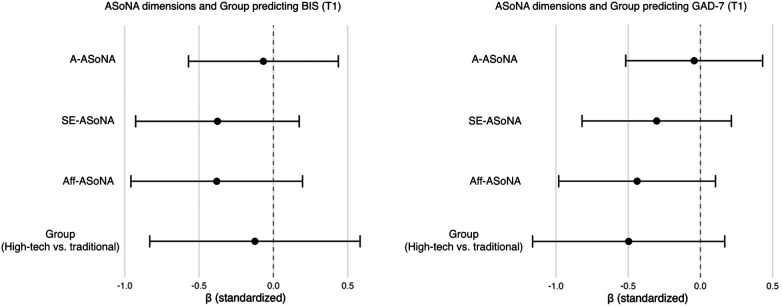
Standardized regression coefficients for BIS and GAD-7 at *T*_1_. Forest plots showing standardized *β* coefficients and 95% confidence intervals for models including ASoNA dimensions and treatment group (high-tech vs. traditional rehabilitation). A-ASonA, Antecedents; SE-ASonA, self-efficacy; Aff-ASonA, positive and negative affectivity.

Multiple regression analyses, with the ASoNA dimensions and Group RCT entered as predictors, confirmed significant models for GAD-7 (*F*_*(*4,16)_ = 7.22, *p* = .002, *R²* = .64) and BIS (*F*_(4,16)_ = 5.90, *p* = .004, *R²* = .60). Standardized regression coefficients for BIS and GAD-7 at T1 are illustrated in Figure 4.

Exploratory correlational analyses were conducted to examine associations between TAPES dimensions and post-treatment psychological and functional outcomes. The TAPES dimensions, including general adjustment (A.G. TAPES), social adjustment (A.S. TAPES), and adjustment to limitations (A.L. TAPES), measured at T1 showed a pattern of significant associations.

General adjustment (A.G. TAPES) was negatively correlated with depressive symptoms (PHQ-9) (*ρ* = −.603, *p* = .004), anxiety symptoms (GAD-7) (*ρ* = −.549, *p* = .010), and body image perception (BIS) (*ρ* = −.555, *p* = .009), and positively correlated with perceived quality of life (EQ-VAS) (*ρ* = .500, *p* = .021) and prosthetic mobility/functionality (AMP) (*ρ* = .452, *p* = .040).

Social adjustment (A.S. TAPES) was negatively correlated with depressive symptoms (PHQ-9) (*ρ* = −.594, *p* = .004) and body image perception (BIS) (*ρ* = −.558, *p* = .009), and positively correlated with perceived quality of life (EQ-VAS) (*ρ* = .598, *p* = .004).

Adjustment to limitations (A.L. TAPES) was positively correlated with body image perception (BIS) (*ρ* = .645, *p* = .002). Total prosthetic satisfaction (tot TAPES) was positively correlated with functional independence (FIM) (*ρ* = .509, *p* = .018) and negatively correlated with body image perception (BIS) (*ρ* = −.580, *p* = .006).

Exploratory regression analyses showed that total prosthetic satisfaction (tot TAPES) was significantly associated with functional independence (FIM) scores at T1 (*β* = .56, *p* = .019, *R*^2^ = .30), while its association with independence in activities of daily living (Barthel Index) showed a trend toward significance (*β* = .45, *p* = .052, *R*^2^ = .28).

Post-hoc power and sensitivity analyses were conducted to further contextualize the statistical findings. *post-hoc* power analyses indicated high observed power for some significant outcomes, particularly FIM, AMP, and PHQ-9. However, given the small sample size, these estimates should be interpreted cautiously. Sensitivity analysis showed that, with *n* = 21, *α* = .05, and power = .80, the study was sensitive to medium-to-large effects (*δ* ≥ 0.63). Bootstrap analyses (5,000 resamples) and leave-one-out procedures were used as robustness checks and supported the stability of the main regression coefficients and correlations. Sensitivity analysis for between-group comparisons indicated that, with 11 participants in the traditional group and 10 in the high-technology group, only large effect sizes (*δ* ≥ 0.89) were statistically detectable.

## 4 Discussion

The findings of the present study suggest that multidisciplinary rehabilitation may be associated with meaningful improvements in both functional and psychological domains.

It should be noted that recruitment was relatively homogeneous with respect to the time elapsed between surgery and the beginning of the prosthetic fitting phase, as all patients entered rehabilitation after surgical wound healing within a 2- to 3-month postoperative window. At the same time, the variability in patients’ subjective psychological and clinical characteristics should be considered when interpreting these preliminary findings, as it reflects the heterogeneity typically observed in real-world rehabilitation settings.

The entire sample showed substantial improvements in autonomy and prosthetic mobility, indicating that the first weeks of intensive training represent a particularly favorable phase for functional recovery. These improvements, observed in both groups, suggest that traditional rehabilitation provides a solid foundation of intervention, while the integration of high-technology tools may be associated with more fine-grained aspects of the adaptation process (10, 36).

The absence of a main effect of group should be interpreted cautiously, given the exploratory nature of the study and the limited sample size. Therefore, the present findings do not allow definitive conclusions regarding differences between rehabilitation approaches. The associations between emotional and functional changes further highlight the complexity of the readjustment process following amputation. Reductions in anxiety were consistently associated with reductions in depressive symptoms, suggesting a possible relationship between emotional regulation and psychological well-being in the post-rehabilitation phase (8, 9). In the traditional rehabilitation group, this pattern was accompanied by higher perceived quality of life, suggesting that a more predictable structured and therapeutic setting may be associated with greater emotional stability (11). In the high-technology group, by contrast, the associations among body image, functional autonomy, and subjective well-being may reflect the role of active engagement with technology in shaping bodily self-perception and perceived functional mastery (15, 16).

The role of baseline psychological resources, as assessed through the ASoNA dimensions, also emerged as relevant. Acceptance, self-efficacy, and affective regulation at baseline were associated with post-treatment psychological and functional outcomes. These findings suggest that initial psychological resources may represent facilitating factors in the rehabilitation process, potentially providing a motivational and emotional basis that supports the reconstruction of bodily identity (8, 11). Similarly, the correlation patterns observed in TAPES dimensions support the view that prosthetic adaptation is a multidimensional process integrating emotional, social, and bodily components (8, 12). Within this framework, overall prosthetic satisfaction may represent a global indicator of rehabilitation quality and the recovery of body image in daily activities and social participation (13).

Overall, the implementation of high-technology rehabilitation did not result in statistically detectable advantages over traditional rehabilitation in the primary outcomes assessed. However, exploratory association patterns observed within the technology-enhanced group suggest that this approach may be related to specific psychological and perceptual aspects of the adaptation process (14, 15). These observations should be considered hypothesis-generating and require confirmation in larger studies.

Taken together, these findings suggest that prosthetic adaptation may be influenced by the interaction of motor abilities, psychological processes, and body representations, and that technological integration may be associated with improvements in specific aspects of this pathway, especially among patients with stronger psychological resources or greater sensitivity to visual and proprioceptive feedback (16, 36).

However, several limitations should be considered when interpreting these findings. First, the multidimensional and complex nature of prosthetic rehabilitation represents an inherent challenge, as the combined effects of physiatric, psychological, motor, and technological interventions make it difficult to isolate the specific contribution of each treatment component (36). Although information regarding ongoing psychological counseling, psychotherapy, and psychiatric treatment was collected at study entry and no participant reported receiving external psychological or psychiatric support, unmeasured psychosocial factors may still have influenced rehabilitation outcomes. Additionally, the relatively short follow-up period, limited to inpatient rehabilitation, does not allow conclusions regarding the long-term stability of improvements or the trajectory of prosthetic adaptation after discharge (10). The predominant use of self-report measures may also introduce subjective bias, while the lack of assessment of perceptions of technology in the control group limits direct comparison of rehabilitation experiences. Finally, the study was conducted in a single rehabilitation center with highly standardized protocols, which, while ensuring methodological consistency, may limit the generalizability of the findings (6). The relatively small sample size further reduces statistical power, particularly for between-group comparisons, and prevents definitive conclusions regarding the comparative effectiveness of the two rehabilitation approaches. Consequently, the study was not designed nor sufficiently powered to establish treatment efficacy or equivalence between rehabilitation approaches, and all between-group findings should be interpreted as exploratory. Although prosthetic components were standardized across participants and the postoperative rehabilitation window was relatively homogeneous, exact individual-level data on time since initial prosthetic fitting, daily prosthesis use before admission, and subjective stage of prosthetic adaptation were not available. In a small sample, residual variability in these factors may have influenced baseline functional status and the trajectory of improvement during rehabilitation. A further limitation concerns the composition of the sample, which included exclusively male participants. While this reflects the epidemiological characteristics of non-traumatic lower-limb amputation, more prevalent among older men with vascular disease or diabetes, it also restricts the generalizability of the findings to a broader amputee population. In particular, the psychological patterns observed in this study, including those related to body image, self-efficacy, and emotional regulation, may differ in other groups such as women, younger individuals, or patients with traumatic amputation, in whom the experience of limb loss and prosthetic integration is shaped by different psychosocial dynamics. Gender has been identified as a relevant factor in psychological adjustment following amputation. Previous research suggests that women may report higher levels of body image disturbance and social anxiety, whereas men may be more likely to discontinue prosthesis use due to functional dissatisfaction or discomfort. In this context, the exclusive inclusion of male participants may provide only a partial representation of the psychological processes underlying prosthetic adaptation, especially for constructs that are closely related to body perception and social functioning. These considerations are particularly relevant when interpreting findings related to psychological variables and their association with functional outcomes. The absence of gender variability in the present sample may have limited the ability to capture a broader range of adaptation profiles and coping strategies. Future research should therefore aim to include more diverse and stratified samples, allowing for the examination of gender-related differences and their interaction with both psychological resources and technological rehabilitation approaches. In addition, extending investigation to different clinical populations, including traumatic and upper-limb amputees, may help to further clarify the role of psychological and technological factors in prosthesis use and long-term adaptation.

These findings suggest several directions for future research and clinical development. Studies with larger, more diverse, and stratified samples are needed to better account for individual and contextual variability, including gender, age, and social support, which are known to influence rehabilitation outcomes and adaptation trajectories (6, 10). Longitudinal follow-up designs would also be valuable to examine the stability of functional and psychological changes over time and to better understand long-term prosthetic integration.

From a clinical perspective, the results suggest that psychological dimensions such as self-efficacy, acceptance, emotional regulation, and body image may represent relevant factors in the rehabilitation process, in line with previous literature on psychological determinants of prosthetic adaptation (8, 11). The integration of targeted psychological interventions early in the rehabilitation pathway may therefore support more comprehensive and individualized care.

From a technological perspective, future research should aim to better distinguish the motivational effects of technology from those related to sensorimotor integration, as technology-based rehabilitation may support engagement and motor learning through augmented feedback mechanisms (14, 15).

Overall, the present findings suggest that multidisciplinary rehabilitation may be associated with improvements in functional autonomy, prosthetic mobility, and psychological well-being in patients with transtibial amputation due to vascular or diabetic causes. The observed gains in independence and mobility suggest that the inpatient rehabilitation period may represent a sensitive phase for motor skill acquisition and prosthetic integration. Although no clear differences between traditional and high-technology rehabilitation were observed in primary functional outcomes, technological tools may be associated with specific psychological dimensions, particularly those related to body image perception and emotional regulation. The observed variability in these characteristics and their association with changes in body image suggest that personality may be related to how patients process limb loss and integrate the prosthesis into their body schema. These findings may have implications for identifying patients who could benefit from more targeted psychological support. Similarly, baseline psychological resources were associated with post-intervention outcomes, including anxiety, depression, body image, and functional recovery. These findings suggest that the psychological condition at admission may play a relevant role in shaping the rehabilitation trajectory. Moreover, final indicators of prosthetic adjustment highlight that successful rehabilitation may depend not only on motor abilities but also on the interaction of emotional, relational, and identity-related factors.

Taken together, these results support the view that prosthetic adaptation is a multidimensional process involving motor functioning, psychological experiences, and body perception. In this exploratory sample, technology-enhanced rehabilitation was not associated with superior physical outcomes compared with traditional rehabilitation. However, exploratory findings suggest potential associations with specific psychological and perceptual aspects of the adaptation process, particularly those related to body integration and emotional regulation. The integration of psychological assessment, individualized interventions, and technological tools may therefore represent a promising direction for the development of more personalized rehabilitation pathways, although these preliminary observations require confirmation in adequately powered studies. Although preliminary, these findings highlight the potential value of systematically including psychological assessments within clinical protocols for amputee patients. Considering these factors may support the identification of different rehabilitation profiles, guide treatment selection, and contribute to the development of more tailored motor–psychological interventions. Future studies with larger samples and extended follow-up periods will be necessary to further clarify these associations and to better understand the role of technology in supporting long-term adaptation.

## Data Availability

The raw data supporting the conclusions of this article will be made available by the authors, without undue reservation.
